# The mediating role of the aggregate index of systemic inflammation between childhood emotional neglect and depressive severity in major depressive disorder: a cross-sectional study

**DOI:** 10.3389/fpsyt.2026.1856338

**Published:** 2026-06-10

**Authors:** Yongqi Ding, Xiaomei Dong, Qi Wang, Tianchao Xu

**Affiliations:** 1 Department of Psychiatry and Psychology, General Hospital of Northern Theater Command, Shenyang, Liaoning, China; 2 School of Medical Humanities, China Medical University, Shenyang, Liaoning, China

**Keywords:** childhood trauma, emotional neglect, major depressive disorder, mediation model, the aggregate index of systemic inflammation

## Abstract

**Objective:**

This cross-sectional study aimed to explore the associations among childhood trauma, inflammation and depressive severity, with a particular focus on the mediating role of a peripheral blood inflammatory marker (the Aggregate Index of Systemic Inflammation, AISI) in the relationship between childhood emotional neglect and depressive severity.

**Methods:**

A total of 236 patients with major depressive disorder (MDD) were enrolled in this study. All participants completed the Childhood Trauma Questionnaire (CTQ) and the 24-item Hamilton Depression Rating Scale (HAMD-24), and underwent routine blood tests. Using Hierarchical regression analysis, after controlling for fixed covariates (age, sex, education level, etc.), separate models were built for each indicator to compare their predictive ability for HAMD-24 scores. The PROCESS mediation model was employed to examine the mediating role of AISI in the relationship between childhood emotional neglect and depressive severity.

**Results:**

MDD patients with a history of childhood trauma exhibited significantly higher levels of inflammatory markers and depressive severity than those without such a history. Among the dimensions of childhood trauma, emotional neglect exhibited the strongest predictive power for HAMD-24 scores (ΔR² = 0.111, ΔP < 0.001, AIC = 1691.663), and among inflammatory markers, AISI demonstrated the strongest predictive power for HAMD-24 scores (ΔR² = 0.230, ΔP < 0.001, AIC = 1654.19). Emotional neglect was positively correlated with AISI (r = 0.213, p < 0.001) and with HAMD-24 scores (r = 0.280, p < 0.001). Furthermore, the inflammatory marker AISI partially mediated the relationship between childhood emotional neglect and depressive severity, with a mediation effect of 28.07%.

**Conclusion:**

Childhood emotional neglect may be associated with depression severity through alterations in systemic inflammatory responses, supporting inflammation as a plausible mediating mechanism in this pathway. While causal relationships cannot be established, these results highlight the potential value of investigating anti-inflammatory strategies as adjunctive treatments for MDD in future prospective studies.

## 1 Introduction

Major depressive disorder (MDD) is a common mental disorder characterized by significant and persistent low mood, anhedonia, and reduced energy, often accompanied by symptoms such as cognitive impairment, autonomic nervous system dysfunction, and sleep disturbances (1, 2). In recent years, its incidence has increased markedly, with a reported prevalence of 7.8% in China (3), imposing a substantial burden on both society and families. Although the pathogenesis of MDD remains unclear, accumulating evidence has established a close association between immune-inflammatory processes and the onset and progression of MDD (4–6), Inflammation may contribute to the development of MDD through multiple pathways, including neuroendocrine dysfunction, disruption of brain neural circuits, impaired synaptic plasticity, and epigenetic modifications.

Among the various risk factors for MDD, childhood trauma (CT) has been confirmed to be closely associated with both the onset and poor prognosis of MDD (7). CT refers to traumatic events experienced during childhood and adolescence that exceed an individual’s coping capacity, including emotional or physical neglect, abuse, and sexual abuse.

Emotional neglect is a subtype of CT, defined as the chronic failure of parents or caregivers to recognize, respond to, and meet a child’s emotional needs, thereby failing to provide the necessary emotional support and psychological care (8). Unlike other types of CT, such as physical abuse and sexual abuse, emotional neglect is more common among adolescents (9), and emotional neglect has been confirmed as a risk factor for MDD in multiple studies (10). Adolescents who are emotionally neglected may develop feelings of insecurity, or perceive themselves as unacceptable and unworthy of being loved by others. These negative feelings may increase the risk of experiencing MDD.

Furthermore, recent studies have found that MDD patients with a history of CT exhibit significant alterations in inflammatory markers (11, 12). Moreover, large-scale cohort studies have revealed that inflammatory markers derived from routine blood parameters—such as the systemic immune-inflammation index (SII) (13, 14), the aggregate index of systemic inflammation (AISI) (13, 15), the systemic inflammation response index (SIRI) (13, 16), the neutrophil-to-lymphocyte ratio (NLR), the platelet-to-lymphocyte ratio (PLR), the derived neutrophil-to-lymphocyte ratio (dNLR) (17), and the aggregated inflammation score (INFLA score) (18)—serve as comprehensive indicators of systemic inflammation and are associated with diverse clinical outcomes in patients with MDD.

AISI is a novel composite inflammatory index derived from routine blood tests that integrates three major pro-inflammatory cell types (platelets, neutrophils, and monocytes) and one key anti-inflammatory regulatory cell type (lymphocytes). It therefore provides a more comprehensive reflection of the pro-inflammatory versus anti-inflammatory balance in the body, representing overall systemic inflammatory levels. Compared with other inflammatory markers (e.g., SII, SIRI, etc.), AISI theoretically captures a more complete spectrum of inflammatory response dimensions, offering unique advantages (19).

Although previous studies have separately investigated the associations between CT and systemic inflammation (20, 21), as well as between inflammation and the onset and progression of MDD (22, 23), several limitations remain. These include the inclusion of non-first-episode MDD patients, insufficient adjustment for confounding factors, and a failure to distinguish between different subtypes of CT. Furthermore, studies that simultaneously examine CT, inflammatory indicators, and depression severity in patients with MDD remain limited. Therefore, the present study distinguished between first-episode MDD patients with CT (CT group) and without CT (NON-CT group), measured systemic inflammatory levels using routine blood parameters, and explored the mediating role of the aggregate index of systemic inflammation (AISI) in the relationship between childhood emotional neglect and depression severity. This study aims to clarify the mechanisms underlying the contribution of childhood emotional neglect to MDD and to provide novel insights into the pathogenesis of MDD.

## 2 Materials and methods

### 2.1 Research design

This study employed a cross-sectional design to measure and analyze childhood trauma, inflammation levels, and depression severity in outpatients with major depressive disorder (MDD) recruited from the General Hospital of Northern Theater Command and the Second Hospital of Lanzhou University between August 2024 and November 2025.

### 2.2 Participants

All participants in this study were from the General Hospital of Northern Theater Command and the Second Hospital of Lanzhou University. The General Hospital of Northern Theater Command is located in Shenyang, Liaoning Province, and the Second Hospital of Lanzhou University is located in Lanzhou, Gansu Province. Both hospitals are Class A tertiary general hospitals, open to patients from all regions, with multiple clinical departments and a broad range of services. The inclusion and exclusion criteria of this study are as follows:

Inclusion criteria: (1) age ranges from 18 to 60; (2) first-episode and drug-naïve; (3) depressive symptoms persisting for ≥ 2 weeks and 24-item Hamilton Depression Rating Scale (HAMD-24) scores ≥ 20; (4) ability to complete the scale assessments.
Exclusion criteria: (1) uncontrolled chronic diseases (e.g., hypertension, diabetes mellitus); (2) comorbid other severe psychiatric disorders (e.g., schizophrenia, bipolar disorder); (3) acute or chronic infection; (4) thyroid disorders (24); (5) recent trauma or surgery; (6) pregnancy or lactation; (7) female patients who were in the menstrual cycle at enrollment prior to enrollment.

Each participant was evaluated by a qualified psychiatrist. The screening process included a psychiatric interview and a standardized diagnostic interview based on the DSM-5.

This study was approved by the Medical Ethics Committees of the General Hospital of Northern Theater Command and the Second Affiliated Hospital of Lanzhou University. All participants provided written informed consent.

### 2.3 Study tool

Participants enrolled in the study were required to provide general information, complete the childhood trauma questionnaire, HAMD-24 scores, and provide fasting peripheral blood samples for routine blood tests.

#### 2.3.1 General information questionnaire

Trained psychiatrists explained the study procedures, objectives, and significance to the participants. After obtaining written informed consent, general demographic and clinical information—including age, sex, height, weight, marital status, education level, disease duration, underlying diseases, and psychiatric history—was collected using a self-designed questionnaire and completed through patient self-report.

#### 2.3.2 Childhood trauma questionnaire

The Childhood Trauma Questionnaire (CTQ) was used to screen for CT. The scale consists of 28 items grouped into five subscales: physical abuse, physical neglect, sexual abuse, emotional abuse, and emotional neglect. Higher scores indicate greater severity of trauma (25). The cut-off scores for defining CT were as follows: physical abuse ≥ 10, physical neglect ≥ 10, sexual abuse ≥ 8, emotional abuse ≥ 13, and emotional neglect ≥ 15. Participants who met any of these criteria were classified as having a history of CT; those who met none were classified as having no such history.

#### 2.3.3 24-item hamilton depression rating scale

Depressive severity was assessed using the HAMD-24. The scale consists of 24 items, and the total score indicates the severity of depression: higher scores indicate more severe depression, whereas lower scores indicate milder depression. Most items are rated on a 5-point Likert-type scale (0–4), with some items using a 3-point scale (0–2). The scale evaluates seven symptom domains: anxiety-somatization, cognitive disturbance, retardation, despair, weight change, sleep disturbance, and diurnal variation. Total scores range from 0 to 78, with cutoffs defined as follows: < 8 (no depression), 8–20 (mild), 20–35 (moderate), and > 35 (severe) (20). The Cronbach’s α coefficient for the HAMD-24 in this study was 0.86.

#### 2.3.4 Inflammatory marker assessment

Routine blood tests were performed upon participant enrollment. Blood parameters included C-reactive protein (CRP), white blood cell (WBC) count, monocyte (MONO) count, neutrophil (NEUT) count, lymphocyte (LYM) count, and platelet (PLT) count. The General Hospital of Northern Theater Command used the Sysmex XN-9000 Hematology Analyzer (Sysmex Corporation, Kobe, Japan), while the Second Affiliated Hospital of Lanzhou University used the Sysmex XT-1800i Hematology Analyzer (Sysmex Corporation, Kobe, Japan). Although the instrument models differed between the two hospitals, their core detection principles and reagent reaction systems were consistent. Both institutions used only original Sysmex matching reagents, calibrators, and quality control materials, with no mixing of third-party reagents or consumables, thereby avoiding result deviations caused by heterogeneity of the detection systems at the source. Furthermore, both institutions strictly followed the unified standardized operating procedures of Sysmex, ensuring consistency of the testing methods.

Based on the neutrophil, lymphocyte, monocyte, and platelet counts obtained from routine blood tests, the following composite inflammatory indices were calculated: NLR, dNLR, MLR, NMLR, AISI, SII, and SIRI. The indices were defined as follows: NLR=NEUT/LYM、 dNLR=NEUT/(WBC−LYM)、 MLR=MONO/LYM、 SIRI=(NEUT×MONO)/LYM、 NMLR=(NEUT+MONO)/LYM、 SII=(PLT×NEUT)/LYM、 AISI=(NEUT×MONO×PLT)/LYM. In addition, the INFLA score was calculated by integrating CRP, WBC, PLT, and fibrinogen (26, 27). Biomarkers were stratified into scoring tiers based on their decile distribution: values in the upper deciles (7th–10th deciles) were assigned scores from +1 to +4; values in the middle deciles (5th–6th deciles) were assigned a score of 0; and values in the lower deciles (1st–4th deciles) were assigned scores from –4 to –1. The total INFLA score, ranging from –16 to +16, was obtained by summing the scores of the four biomarkers. A higher INFLA score indicates a more pronounced inflammatory status.

### 2.4 Data analysis

Data processing and statistical analyses were performed using SPSS 27.0, SPSSAU (Beijing Qingsi Technology Co., Ltd.), and the PROCESS macro. The significance level was set at α = 0.05, two-tailed. Continuous data that followed a normal distribution according to the Shapiro–Wilk test were described as mean ± standard deviation, and comparisons between two groups were made using the independent samples t-test. Continuous data that did not follow a normal distribution were described as median (interquartile range, IQR) and compared using the Mann–Whitney U test. Categorical data were described as frequencies and percentages and compared using the chi-square test.

Hierarchical regression analysis was conducted. Demographic variables were entered in the first block, and each CT dimension and each inflammatory marker were individually entered in the second block in separate models. This approach aimed to examine the explanatory power of each indicator for HAMD-24 scores after controlling for demographic variables. The most robust indicators were identified by comparing the ΔR² and AIC across these models. Spearman correlation analysis was performed to examine the associations among emotional neglect, AISI, and HAMD-24 scores. Based on previous literature, we hypothesized that emotional neglect is associated with the severity of depression, with AISI playing a mediating role. A mediation model was constructed and tested using the PROCESS macro (Model 4) developed by Hayes, with demographic variables included as control variables. The Bootstrap method with 5,000 resamples was used to obtain 95% confidence intervals (CIs); a mediation effect was considered significant if the 95% CI did not include zero.

## 3 Results

### 3.1 General clinical data of the included patients

A total of 236 patients were enrolled in this study. The prevalence of CT was 69.07%, with 136 cases reporting emotional neglect, 119 cases physical neglect, 65 cases emotional abuse, 22 cases physical abuse, and 26 cases sexual abuse. Based on CTQ scores, participants were divided into a CT group (n = 163) and NON-CTgroup (n = 73). In the CT group, there were 46 males and 117 females, with a median age of 31 years (25, 40) and a median disease duration of 5.5 months (3, 8). In the NON-CT group, there were 28 males and 45 females, with a median age of 31 years (25, 38) and a median disease duration of 6 months (3, 9).

Univariate analysis showed no statistically significant differences between the two groups in age, disease duration, BMI, monthly family income, marital status, or education level (p > 0.05). Statistically significant differences were observed in HAMD-24 scores, NEUT, LYM, AISI, SII, SIRI, NLR, MLR, NMLR, PLR, and INFLA scores (p < 0.05), with the CT group exhibiting higher levels of inflammation and greater depressive severity than the NON-CT group. Detailed demographic and clinical characteristics are summarized in **Table 1**.

**Table 1:** Comprehensive comparison of baseline demographic and clinical data.

Variable	CT group (n = 163)	NON-CT group (n=73)	χ2 (t/z)	p
Sex (n, %)			2.406	0.121
Male	46 (28.22%)	28 (38.36%)		
Female	117 (71.78%)	45 (61.64%)		
Age [years, M (P25, P75)]	31 (25, 40)	31 (25, 38)	-0.058	0.954
Disease duration [months M (P25, P75)]	5.500 (3.0,8.0)	6.000 (3.0,9)	-0.246	0.805
BMI (mean ± SD)	22.84 ± 4.72	22.64 ± 3.18	-0.335	0.738
Monthly family income [10,000 RMB, M (P25, P75)]	1 (0.6, 1.5)	1 (0.6, 2)	-0.965	0.335
Education (n, %)			1.369	0.242
Junior high school or below	52 (31.90%)	29 (39.73%)		
College or above	111 (68.10%)	44 (60.27%)		
Married (n, %)			0.674	0.412
Yes	92 (56.44%)	37 (50.68%)		
No	71 (43.56%)	36 (49.32%)		
HAMD-24 [M (P25, P75)]	36.000 (28.0,42.0)	29.000 (23.0,35.0)	-4.794	<0.001
Inflammatory markers				
CRP [mg/L, M (P25, P75)]	2.320 (1.7,3.5)	2.160 (1.6,3.0)	-1.265	0.206
WBC [×10^9/L, M (P25, P75)]	5.920 (5.1,7.0)	5.800 (4.8,6.8)	-0.950	0.342
MONO [×10^9/L, M (P25, P75)]	0.340 (0.3,0.4)	0.330 (0.3,0.4)	-0.927	0.354
NEUT [×10^9/L, M (P25, P75)]	3.650 (2.9,4.5)	3.350 (2.6,4.1)	-1.977	0.048
LYM [×10^9/L, M (P25, P75)]	1.780 (1.5,2.3)	1.970 (1.6,2.4)	-2.134	0.033
PLT [×10^9/L, M (P25, P75)]	241.000 (209.0,287.0)	244.000 (209.0,287.5)	-0.079	0.937
AISI [M (P25, P75)]	158.787 (104.4,242.1)	120.780 (80.7,196.2)	-2.651	0.008
SII [M (P25, P75)]	471.083 (336.5,649.1)	398.494 (275.2,514.3)	-3.067	0.002
SIRI [M (P25, P75)]	0.676 (0.5,1.0)	0.583 (0.4,0.8)	-2.772	0.006
NLR [M (P25, P75)]	2.023 (1.5,2.6)	1.782 (1.3,2.1)	-3.307	0.001
dNLR [M (P25, P75)]	0.884 (0.9,0.9)	0.881 (0.9,0.9)	-1.474	0.140
MLR [M (P25, P75)]	0.182 (0.1,0.2)	0.164 (0.1,0.2)	-2.340	0.019
NMLR [M (P25, P75)]	2.211 (1.7,2.8)	1.963 (1.4,2.3)	-3.379	0.001
PLR [M (P25, P75)]	139.912 (108.5,169.4)	114.124 (95.7,154.5)	-2.542	0.011
INFLA [M (P25, P75)]	1.000 (-2.0,5.0)	-1.000 (-4.0,3.0)	-2.477	0.013

CT group, MDD patients with CT; NON-CT group, MDD patients without CT; BMI, body mass index; HAMD-24, 24-item Hamilton Depression Scale; CRP, C-reactive protein; WBC, white blood cell; MONO, monocyte; NEUT, neutrophil; LYM, lymphocyte; PLT, platelet; AISI, aggregate index of systemic inflammation; SII, systemic immune-inflammation index; SIRI, systemic inflammation response index; NLR, neutrophil-to-lymphocyte ratio; dNLR, derived neutrophil-to-lymphocyte ratio; MLR, monocyte-to-lymphocyte ratio; NMLR, neutrophil-monocyte to lymphocyte ratio; PLR, platelet-to-lymphocyte ratio; INFLA, aggregated inflammation score.

### 3.2 Regression analysis

As shown in **Table 2**, after controlling for illness duration, sex, education level, BMI, marital status, and monthly household income, emotional neglect exhibited the strongest explanatory power among the CT dimensions (Δ*R*² = 0.111, Δ*p* < 0.001, AIC = 1691.663). **Table 3** shows that among the composite inflammatory markers, AISI demonstrated the best independent predictive ability (Δ*R*² = 0.230, Δ*p* < 0.001), and its model also achieved the optimal goodness of fit (AIC = 1654.19).

**Table 2:** Hierarchical regression analysis of the dimensions of childhood trauma predicting HAMD-24 scores.

Index	β (95% CI)	R^2^	ΔR^2^	Δp	AIC
Emotional neglect	0.68 (0.44, 0.92)	0.184	0.111	<0.001	1691.663
Physical abuse	0.89 (0.45, 1.32)	R^2^	0.061	<0.001	1705.650
Emotional abuse	0.39 (0.19, 0.59)	0.131	0.058	<0.001	1706.398
Sexual abuse	0.92 (0.36, 1.48)	0.114	0.041	0.001	1711.021
Physical neglect	0.33 (0.07, 0.60)	0.098	0.025	0.013	1715.259

β, unstandardized regression coefficient; CI, 95% confidence interval; ΔR², increase in R² when the indicator is added to the model containing control variables; Δp, significance level of ΔR²; AIC, Akaike information criterion.

**Table 3:** Hierarchical regression analysis of inflammatory markers predicting HAMD-24 scores.

Index	β (95% CI)	R2	ΔR2	Δp	AIC
AISI	0.03(0.02, 0.04)	0.304	0.230	<0.001	1654.193
SII	7.32(5.51, 9.14)	0.256	0.183	<0.001	1669.717
SIRI	0.011(0.008,0.014)	0.273	0.199	<0.001	1664.464
NLR	2.70(1.95, 3.44)	0.238	0.165	<0.001	1675.358
dNLR	2.87(2.07, 3.67)	0.074	0.001	0.682	1721.486
MLR	2.87(2.07, 3.67)	0.219	0.145	<0.001	1681.424
NMLR	-1.09(-6.34,4.16)	0.242	0.168	<0.001	1674.417

β, unstandardized regression coefficient; CI, 95% confidence interval; ΔR², increase in R² when the indicator is added to the model containing control variables; Δp, significance level of ΔR²; AIC, Akaike information criterion. AISI, aggregate index of systemic inflammation; SII, systemic immune-inflammation index; SIRI, systemic inflammation response index; NLR, neutrophil-to-lymphocyte ratio; dNLR, derived neutrophil-to-lymphocyte ratio; MLR, monocyte-to-lymphocyte ratio; NMLR, neutrophil-monocyte to lymphocyte ratio.

### 3.3 Correlation analysis

The regression results showed that both emotional neglect and AISI significantly positively predicted HAMD-24 scores. Based on prior research, AISI was selected as the mediating variable for inclusion in the mediation model. Spearman correlation analysis was conducted to examine the relationships among emotional neglect scores, HAMD-24 scores, and AISI. As shown in **Table 4**, emotional neglect scores, AISI, and HAMD-24 scores were all significantly intercorrelated.

**Table 4:** Spearman correlation analysis.

Category	Emotional neglect	AISI	HAMD-24
Emotional neglect	1		
AISI	0.213***	1	
HAMD-24	0.280***	0.332***	1

* *p* < 0.05 ** *p* < 0.01 *** *p* < 0.001; AISI, aggregate index of systemic inflammation.

### 3.4 Mediation analysis

**Figure 1:**
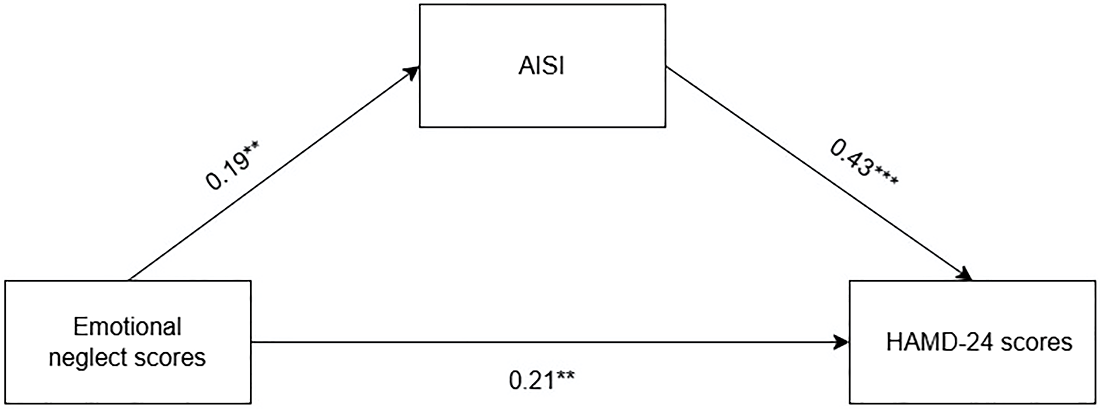
Path diagram of the mediation model. Mediating effect model of EN and AISI on depression severity in patients with MDD. *p < 0.05; **p < 0.01; ***p < 0.001.

The analysis results are presented in **Table 5**. Both EN scores and AISI exhibited significant positive predictive effects on HAMD-24 scores. The bootstrap 95% CI for the indirect effect did not include zero (0.064, 0.213) (**Table 6**), indicating a significant indirect effect. The path diagram of the mediation model is shown in **Figure 1**. AISI partially mediated the relationship between emotional neglect and depression severity, with the indirect effect accounting for 28.07% of the total effect.

**Table 5:** Model regression analysis.

Model	Predictor	Outcome	β	*t*	*p*	95% CI
Model 1	Emotional neglect	AISI	0.189	2.946	0.004	[1.672, 8.421]
Model 2	Emotional neglect	HAMD-24	0.210	3.683	<0.001	[0.153, 0.503]
	AISI		0.434	7.593	<0.001	[0.019, 0.032]

CI, Confidence Interval; AISI, aggregate index of systemic inflammation.

**Table 6:** Analysis of model mediating effect.

Effect	Effect size	SE	95% CI	Proportion of total effect
Total effect	0.456	0.098	[0.264, 0.648]	
Direct effect	0.328	0.089	[0.153, 0.503]	71.93%
Indirect effect	0.128	0.038	[0.064, 0.213]	28.07%

SE, standard error; CI, Confidence Interval.

## 4 Discussion

### 4.1 CT exacerbates inflammatory response and depression severity

Compared with patients without a history of CT, those who experienced CT exhibited higher levels of inflammation and more severe depressive symptoms. Childhood is a sensitive period for the development of the nervous and immune systems, and prolonged exposure to stressful environments during this period may lead to immune dysregulation. According to the neuroimmune hypothesis, CT can induce dysfunction of the hypothalamic-pituitary-adrenal (HPA) axis, resulting in abnormal glucocorticoid levels and glucocorticoid receptor resistance, which in turn promote the release of pro-inflammatory cytokines (28). Furthermore, HPA axis dysfunction caused by CT may lead to abnormal stress responses in adulthood, further increasing the risk of chronic inflammation (29).

CT has been recognized as one of the significant risk factors for the onset of MDD (30, 31) and is closely associated with depression severity, disease course, and prognosis (32). Consistent with previous studies, the present study found that MDD patients with a history of CT a had more severe depressive symptoms than those without such a history. From a neurobiological perspective, CT may lead to HPA axis dysfunction, inducing a low-grade chronic inflammatory state, reduced hippocampal volume, amygdala hyperactivation, and increased individual vulnerability to stress. From a psychosocial perspective, CT may contribute to the formation of negative core beliefs about “self, the world, and the future,” and the development of maladaptive emotion regulation strategies, making individuals more prone to excessive internal attribution and catastrophizing in response to negative life events, thereby exacerbating depression severity.

### 4.2 Analysis of factors influencing depression severity

The results of hierarchical regression analyses showed that, after controlling for demographic variables, emotional neglect demonstrated the strongest independent explanatory power for HAMD-24 scores among the five CT dimensions, and AISI showed the strongest independent explanatory power among the composite inflammatory markers.

Among the 236 MDD patients enrolled in this study, the prevalence of CT was 69.07%, which was slightly lower than the 75.6% reported by Negele et al. (33). CT was more prevalent in females than in males (34). The CTQ evaluates five dimensions of CT: emotional abuse, physical abuse, sexual abuse, emotional neglect, and physical neglect. Previous studies have strongly supported the profound impact of childhood abuse (i.e., emotional abuse, physical abuse, sexual abuse) on depression (35, 36). However, the present study found that emotional neglect contributed to depression severity to a similar extent as traditional childhood abuse dimensions. Childhood abuse involves episodic events that are easily identifiable and recalled. In contrast, emotional neglect represents a chronic, insidious trauma characterized by the persistent failure of caregivers to provide adequate emotional responses, attention, and support. This “absence” is often internalized as normal and is not easily recognized as trauma, yet its cumulative effects exert profound influences on psychological development (37).

AISI is a composite inflammatory index derived from routine complete blood counts, reflecting systemic immune-inflammatory status, and has been widely used as a peripheral biomarker for psychiatric disorders such as MDD. Studies have shown that AISI levels are significantly higher in patients with MDD than in healthy controls, suggesting its potential as a diagnostic biomarker (38). Peripheral inflammatory signals can propagate to the central nervous system, activating microglia and leading to neuroinflammation and disturbances in neurotransmitter metabolism, which may clinically manifest as depressive symptoms such as anhedonia and psychomotor retardation.

### 4.3 Correlation analysis of emotional neglect, AISI, and depression severity

The present study found that emotional neglect, AISI, and depression severity were all significantly correlated with each other. The positive correlation between emotional neglect and depressive severity was consistent with the findings of previous studies (39, 40): chronic lack of emotional response and support during childhood may be associated with impaired emotion regulation capacity, reinforced negative cognitive schemas, and increase vulnerability to stress potential; these factors might accompany an elevated risk for the onset and persistence of MDD.

The positive correlation between emotional neglect and AISI suggests that early psychological trauma may have a profound impact on physiological systems — a lack of adequate emotional support and responsiveness during childhood may be associated with persistent activation of the hypothalamic-pituitary-adrenal (HPA) axis and a chronic low-grade inflammatory state, as reflected in changes in peripheral blood composite inflammatory indices such as AISI (38).

AISI was positively correlated with depression severity. The study by Sălcudean et al. systematically elaborated on the bidirectional relationship between neuroinflammation and MDD: peripheral inflammatory signals may influence central nervous system activity through the blood-brain barrier, the vagus nerve, and surface receptors on cerebrovascular endothelial cells, and have been linked to the occurrence of neuroinflammation, interference with monoamine neurotransmitter metabolism, and reduced neuroplasticity; these processes may accompany the development and maintenance of depressive symptoms. Meanwhile, depression itself may also be associated with an exacerbation of inflammatory responses, forming a potential reciprocal influence loop (41).

### 4.4 The mediating role of AISI between emotional neglect and depression severity

By exploring the potential mechanisms underlying the association between childhood emotional neglect and depressive severity in patients with MDD, the present study found that AISI played a partial mediating role in this relationship. This finding suggests that emotional neglect may be directly associated with depressive severity, and may also be indirectly related via inflammatory levels as reflected by AISI. These observations provide new empirical support for the biopsychosocial model systematically proposed by Engel (1977), which emphasizes that human health and disease arise from the interaction of biological, psychological, and social factors, rather than being determined by any single dimension alone.

Previous studies have predominantly examined the relationship between emotional neglect and depression from cognitive perspectives (e.g., psychological resilience, self-esteem, rumination) (42, 43). In contrast, the results of the present study suggest that emotional neglect may not only be associated with mental health through cognitive-emotional pathways, but may also involve the biological system (immune-inflammatory pathways) and be linked to emotional states via neuroimmune mechanisms. This multi-channel pattern of associations indicates that the relationship between emotional neglect and depressive severity may have an integrated, multi-level nature.

As a chronic, cumulative “trauma of omission”, emotional neglect may be associated with persistent physiological alterations through a “biological embedding” mechanism, such as disrupted development of central neural networks, dysfunction of the hypothalamic-pituitary-adrenal (HPA) axis, and the induction of a chronic low-grade inflammatory state (44, 45). In addition, inflammatory sensitization mechanisms may lead affected individuals to exhibit exaggerated inflammatory responses to stressors in adulthood, manifesting as abnormally elevated peripheral blood inflammatory indices such as AISI.

Peripheral inflammatory signals may then be transmitted to the central nervous system via the vagus nerve, blood-brain barrier, and other routes, where they may be associated with microglial activation, interference with monoamine neurotransmitter metabolism, and impairment of neuroplasticity—processes that might further contribute to depressive symptoms (45). Overall, the association between childhood emotional neglect and depressive severity may involve multiple pathways and levels, including not only cognitive-emotional pathways but also biological pathways of neuroimmune interaction.

The composite inflammatory marker AISI may serve as a potential peripheral blood biomarker for identifying individuals with a history of childhood emotional neglect who are at higher risk of greater depression severity, thereby aiding in early screening (38). The partial mediating effect of AISI suggests that there may be dual intervention targets at both the psychological level (e.g., trauma-focused therapy, emotion regulation training) and the physiological level (e.g., lifestyle modifications, anti-inflammatory diet, or targeted anti-inflammatory strategies validated by future research). Furthermore, the findings underscore the importance of early identification of childhood emotional neglect—even in the absence of overt abuse, prolonged emotional deprivation may partially affect mental health through neuroimmune pathways.

This study reveals that childhood emotional neglect, a form of “hidden trauma,” is associated with depression severity not only through psychological and cognitive pathways but also potentially through biological pathways such as inflammation. These findings encourage the public, educators, and healthcare professionals to re-examine the long-term health consequences of emotional neglect and to promote greater attention and responsiveness to children’s emotional needs at the family, school, and community levels, thereby reducing the occurrence of emotional neglect at its source. Meanwhile, inflammatory indices derived from routine blood tests, such as AISI, show promise as low-cost potential screening tools. They may help identify high-risk individuals in primary care or school health examinations, enabling early warning and tiered intervention, and ultimately alleviating personal suffering, family burden, and the long-term strain on societal healthcare resources.

## 5 Conclusion

In summary, the present study draws the following conclusions: (1) In patients with MDD, a history of CT is often associated with more severe inflammatory responses and greater depression severity. (2) emotional neglect, AISI, and depression severity are all significantly positively correlated with each other in MDD patients. (3) AISI partially mediates the relationship between emotional neglect and depression severity.

From a biopsychosocial integrative perspective, this study found a significant association between childhood emotional neglect, AISI levels, and depression severity. These preliminary findings suggest that AISI may be a potential intermediate variable in the relationship between childhood emotional neglect and depression, and inflammatory response may be one possible biological link between early adverse experiences and later psychopathological outcomes. This study generates hypotheses regarding the immune-psychological mechanisms of MDD and provides preliminary insights for future research on interventions targeting inflammatory pathways.

## 6 Limitations

Several limitations of this study should be acknowledged. First, the cross-sectional design precludes the establishment of causal relationships among emotional neglect, systemic inflammation (AISI), and depression severity. The identified mediating pathway only reflects statistical associations rather than definitive causal links; thus, longitudinal studies are warranted to verify the directionality of these relationships. Second, the study population was geographically restricted, as only outpatients from two hospitals were included, and potential regional and cultural influences were not accounted for. Future studies should enroll participants from diverse regions to validate the generalizability of the present findings. Third, childhood emotional neglect was evaluated using a self-reported questionnaire, which did not capture characteristics such as duration, severity, or age at onset—factors that may have differential effects on inflammatory status and depression severity.

## Data Availability

The raw data supporting the conclusions of this article will be made available by the authors, without undue reservation.

## References

[1] Belmaker RH Agam G . Major depressive disorder. N Engl J Med. (2008) 358:55–68. doi: 10.1056/nejmra073096 18172175

[2] Qiu M Zhang C Zhang H Chen H Lei Y Li P . Retrospective evaluation of novel serum inflammatory biomarkers in first-episode psychiatric disorders: diagnostic potential and immune dysregulation. Front Psychiatry. (2024) 15:1442954. doi: 10.3389/fpsyt.2024.1442954 39722850 PMC11668741

[3] Lu J Xu X Huang Y Li T Ma C Xu G . Prevalence of depressive disorders and treatment in China: a cross-sectional epidemiological study. Lancet Psychiatry. (2021) 8:981–90. doi: 10.1016/S2215-0366(21)00251-0 34559991

[4] Beurel E Toups M Nemeroff CB . The bidirectional relationship of depression and inflammation: double trouble. Neuron. (2020) 107:234–56. doi: 10.1016/j.neuron.2020.06.002 32553197 PMC7381373

[5] Miller AH Raison CL . The role of inflammation in depression: from evolutionary imperative to modern treatment target. Nat Rev Immunol. (2016) 16:22–34. doi: 10.1038/nri.2015.5 26711676 PMC5542678

[6] Prakash S Bhatia D . The multifaceted pathophysiology of major depressive disorder: integrating neurobiology, genetics, and systems-level perspectives. Curr Neurovasc Res. (2026) 23:5–19. doi: 10.2174/0115672026415993251226184643 41735218

[7] Nelson J Klumparendt A Doebler P Ehring T . Childhood maltreatment and characteristics of adult depression: meta-analysis. Br J Psychiatry. (2017) 210:96–104. doi: 10.1192/bjp.bp.115.180752 27908895

[8] Kumari V . Emotional abuse and neglect: time to focus on prevention and mental health consequences. Br J Psychiatry. (2020) 217:597–9. doi: 10.1192/bjp.2020.154 32892766 PMC7589986

[9] Brown S Fite PJ Stone K Richey A Bortolato M . Associations between emotional abuse and neglect and dimensions of alexithymia: the moderating role of sex. Psychol Trauma Theory Res Pract Policy. (2018) 10:300–8. doi: 10.1037/tra0000279 28414491 PMC5645215

[10] Fung HW Chung HM Ross CA . Demographic and mental health correlates of childhood emotional abuse and neglect in a Hong Kong sample. Child Abuse Negl. (2020) 99:104288. doi: 10.1016/j.chiabu.2019.104288 31821980

[11] Schirmer ST Beckmann FE Gruber H Schlaaff K Scheermann D Seidenbecher S . Decreased functional connectivity in patients with major depressive disorder and a history of childhood traumatization through experiences of abuse. Behav Brain Res. (2023) 437:114098. doi: 10.1016/j.bbr.2022.114098 36067949

[12] Brown M Worrell C Pariante CM . Inflammation and early life stress: an updated review of childhood trauma and inflammatory markers in adulthood. Pharmacol Biochem Behav. (2021) 211:173291. doi: 10.1016/j.pbb.2021.173291 34695507

[13] Li X Huan J Lin L Hu Y . Association of systemic inflammatory biomarkers with depression risk: results from National Health and Nutrition Examination Survey 2005–2018 analyses. Front Psychiatry. (2023) 14:1097196. doi: 10.3389/fpsyt.2023.1097196 36846218 PMC9945233

[14] Wang M Peng C Jiang T Wu Q Li D Lu M . Association between systemic immune-inflammation index and post-stroke depression: a cross-sectional study of the national health and nutrition examination survey 2005–2020. Front Neurol. (2024) 15:1330338. doi: 10.3389/fneur.2024.1330338 38562426 PMC10984268

[15] Duan J Chen J Xiang Z . The U-shape relationship between the aggregate index of systemic inflammation and depression in American adults: a cross-sectional study. J Affect Disord. (2025) 380:270–8. doi: 10.1016/j.jad.2025.03.139 40147607

[16] Zhou Y Zhang C Li J Zheng Y Xiao S . Systemic inflammation mediates the association between dietary inflammation index and incident anxiety and depression in UK Biobank. J Affect Disord. (2025) 381:205–14. doi: 10.1016/j.jad.2025.03.101 40158861

[17] Cheng Y Wang Y Wang X Jiang Z Zhu L Fang S . Neutrophil-to-lymphocyte ratio, platelet-to-lymphocyte ratio, and monocyte-to-lymphocyte ratio in depression: an updated systematic review and meta-analysis. Front Psychiatry. (2022) 13:893097. doi: 10.3389/fpsyt.2022.893097 35782448 PMC9240476

[18] Gialluisi A Costanzo S Castelnuovo AD Bonaccio M Bracone F Magnacca S . Combined influence of depression severity and low-grade inflammation on incident hospitalization and mortality risk in Italian adults. J Affect Disord. (2021) 279:173–82. doi: 10.1016/j.jad.2020.10.004 33059220

[19] Zengin O Göre B Öztürk O Cengiz AM Güler Kadıoğlu S Asfuroğlu Kalkan E . Evaluation of acute pancreatitis severity and prognosis using the aggregate systemic inflammation index (AISI) as a new marker: a comparison with other inflammatory indices. J Clin Med. (2025) 14:3419. doi: 10.3390/jcm14103419 40429414 PMC12111921

[20] Gill H El-Halabi S Majeed A Gill B Lui LMW Mansur RB . The association between adverse childhood experiences and inflammation in patients with major depressive disorder: a systematic review. J Affect Disord. (2020) 272:1–7. doi: 10.1016/j.jad.2020.03.145 32379599

[21] Baumeister D Akhtar R Ciufolini S Pariante CM Mondelli V . Childhood trauma and adulthood inflammation: a meta-analysis of peripheral C-reactive protein, interleukin-6 and tumour necrosis factor-α. Mol Psychiatry. (2016) 21:642–9. doi: 10.1038/mp.2015.67 26033244 PMC4564950

[22] Debnath M Berk M Maes M . Translational evidence for the inflammatory response system (IRS)/compensatory immune response system (CIRS) and neuroprogression theory of major depression. Prog Neuro-Psychopharmacol Biol Psychiatry. (2021) 111:110343. doi: 10.1016/j.pnpbp.2021.110343 33961966

[23] Réus GZ Manosso L Quevedo J Carvalho AF . Major depressive disorder as a neuro-immune disorder: origin, mechanisms, and therapeutic opportunities. Neurosci Biobehav Rev. (2023) 155:105425. doi: 10.1016/j.neubiorev.2023.105425 37852343

[24] Yang R Li Z Zhu Y Wu Y Lu X Zhao X . Non-linear relationship between TSH and psychotic symptoms on first episode and drug naïve major depressive disorder patients: a large sample sized cross-sectional study in China. BMC Psychiatry. (2024) 24:413. doi: 10.1186/s12888-024-05860-7 38834989 PMC11151505

[25] Bernstein DP Stein JA Newcomb MD Walker E Pogge D Ahluvalia T . Development and validation of a brief screening version of the Childhood Trauma Questionnaire. Child Abuse Negl. (2003) 27:169–90. doi: 10.1016/S0145-2134(02)00541-0 12615092

[26] Bondonno NP Parmenter BH Thompson AS Jennings A Murray K Rasmussen DB . Flavonoid intakes, chronic obstructive pulmonary disease, adult asthma, and lung function: a cohort study in the UK Biobank. Am J Clin Nutr. (2024) 120:1195–206. doi: 10.1016/j.ajcnut.2024.08.032 39222688 PMC11600086

[27] Gialluisi A Santonastaso F Bonaccio M Bracone F Shivappa N Hebert JR . Circulating inflammation markers partly explain the link between the dietary inflammatory index and depressive symptoms. J Inflammation Res. (2021) 14:4955–68. doi: 10.2147/JIR.S312925 34611421 PMC8487281

[28] Holochwost SJ Gomes LA Wylie A Kolacz J . Resting hypothalamic–pituitary–adrenal axis activity in childhood following maltreatment: a meta‐analysis. Dev Psychobiol. (2025) 67:e70022. doi: 10.1002/dev.70022 40007054

[29] Marques-Feixa L Palma-Gudiel H Romero S Moya-Higueras J Rapado-Castro M Castro-Quintas Á . Childhood maltreatment disrupts HPA-axis activity under basal and stress conditions in a dose–response relationship in children and adolescents. Psychol Med. (2023) 53:1060–73. doi: 10.1017/S003329172100249X 34269169 PMC9976019

[30] Gardner MJ Thomas HJ Erskine HE . The association between five forms of child maltreatment and depressive and anxiety disorders: a systematic review and meta-analysis. Child Abuse Negl. (2019) 96:104082. doi: 10.1016/j.chiabu.2019.104082 31374447

[31] Humphreys KL LeMoult J Wear JG Piersiak HA Lee A Gotlib IH . Child maltreatment and depression: a meta-analysis of studies using the Childhood Trauma Questionnaire. Child Abuse Negl. (2020) 102:104361. doi: 10.1016/j.chiabu.2020.104361 32062423 PMC7081433

[32] Lippard ETC Nemeroff CB . The devastating clinical consequences of child abuse and neglect: increased disease vulnerability and poor treatment response in mood disorders. Am J Psychiatry. (2020) 177:20–36. doi: 10.1176/appi.ajp.2019.19010020 31537091 PMC6939135

[33] Negele A Kaufhold J Kallenbach L Leuzinger-Bohleber M . Childhood trauma and its relation to chronic depression in adulthood. Depress Res Treat. (2015) 2015:1–11. doi: 10.1155/2015/650804 26693349 PMC4677006

[34] Young EA Abelson JL Curtis GC Nesse RM . Childhood adversity and vulnerability to mood and anxiety disorders. Depress Anxiety. (1997) 5:66–72. doi: 10.1002/(sici)1520-6394(1997)5:2<66::aid-da2>3.0.co;2-3 9262936

[35] Wang X Ding F Cheng C He J Wang X Yao S . Psychometric properties and measurement invariance of the Childhood Trauma Questionnaire (Short Form) across genders, time points and presence of major depressive disorder among Chinese adolescents. Front Psychol. (2022) 13:816051. doi: 10.3389/fpsyg.2022.816051 35478747 PMC9036057

[36] Zhu Y Wang MJ Crawford KM Ramírez-Tapia JC Lussier AA Davis KA . Sensitive period-regulating genetic pathways and exposure to adversity shape risk for depression. Neuropsychopharmacology. (2022) 47:497–506. doi: 10.1038/s41386-021-01172-6 34689167 PMC8674315

[37] Saladino V Verrastro V Giordano F Calaresi D . The longitudinal effects of emotional neglect and psychoticism on adolescent depression. Psychol Psychother Theory Res Pract. (2026) 99:185–200. doi: 10.1111/papt.70017 41077865

[38] Wang Q Li L Yang B Wang J Wei X Shen Y . The aggregate index of systemic inflammation as peripheral inflammatory markers for first-episode drug-naïve patients with major depressive disorder. J Affect Disord. (2026) 403:121435. doi: 10.1016/j.jad.2026.121435 41707720

[39] Whitaker RC Dearth-Wesley T Herman AN . The association of childhood parental connection with adult flourishing and depressive symptoms. Pediatrics. (2024) 153:e2023064690. doi: 10.1542/peds.2023-064690 38425226 PMC10904890

[40] Zhong X Li S Zhou W . Childhood emotional neglect and depression in emerging adults: exploring the roles of rumination and resilience. Acta Psychol (Amst). (2025) 258:105272. doi: 10.1016/j.actpsy.2025.105272 40639186

[41] Sălcudean A Popovici RA Pitic DE Sârbu D Boroghina A Jomaa M . Unraveling the complex interplay between neuroinflammation and depression: a comprehensive review. Int J Mol Sci. (2025) 26:1645. doi: 10.3390/ijms26041645 40004109 PMC11855341

[42] Ji L . Childhood emotional abuse and depression among Chinese adolescent sample: a mediating and moderating dual role model of rumination and resilience. Child Abuse Negl. (2024) 149:106607. doi: 10.1016/j.chiabu.2023.106607 38154376

[43] Strecker K Sim EJ Woike K Schönfeldt-Lecuona C Radermacher P Karabatsiakis A . Association of the biopsychosocial factors adverse childhood experiences, adult attachment style, emotion regulation, and mitochondrial density in immune cells with major depressive disorder. Neuroimmunomodulation. (2025) 32:110–23. doi: 10.1159/000544833 40159392

[44] Kumsta R . The role of stress in the biological embedding of experience. Psychoneuroendocrinology. (2023) 156:106364. doi: 10.1016/j.psyneuen.2023.106364 37586308

[45] Wang J Xu Q Tang X Hu A Zhang D Liu Z . Neuroimmune programming of childhood trauma: comorbid mechanisms and developmental origins of depression and autoimmune diseases. J Neuroinflamm. (2026) 23:182. doi: 10.1186/s12974-026-03810-6 42001138 PMC13227822

